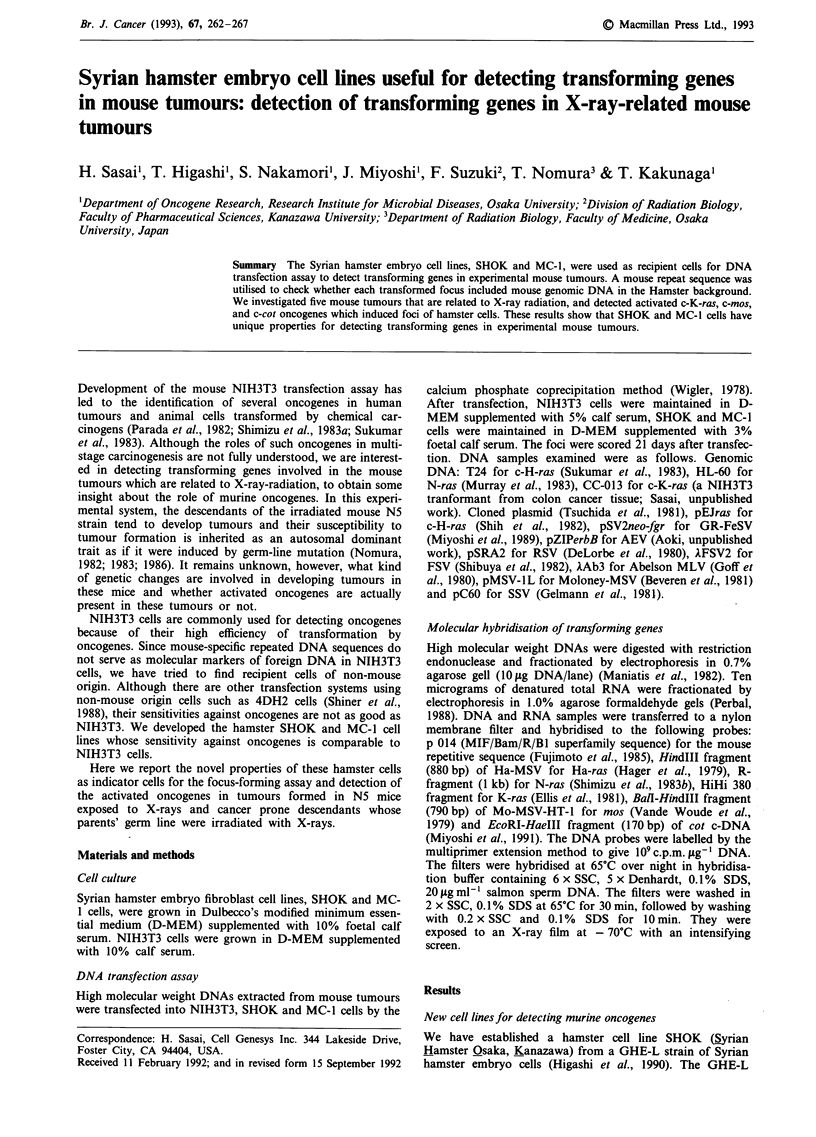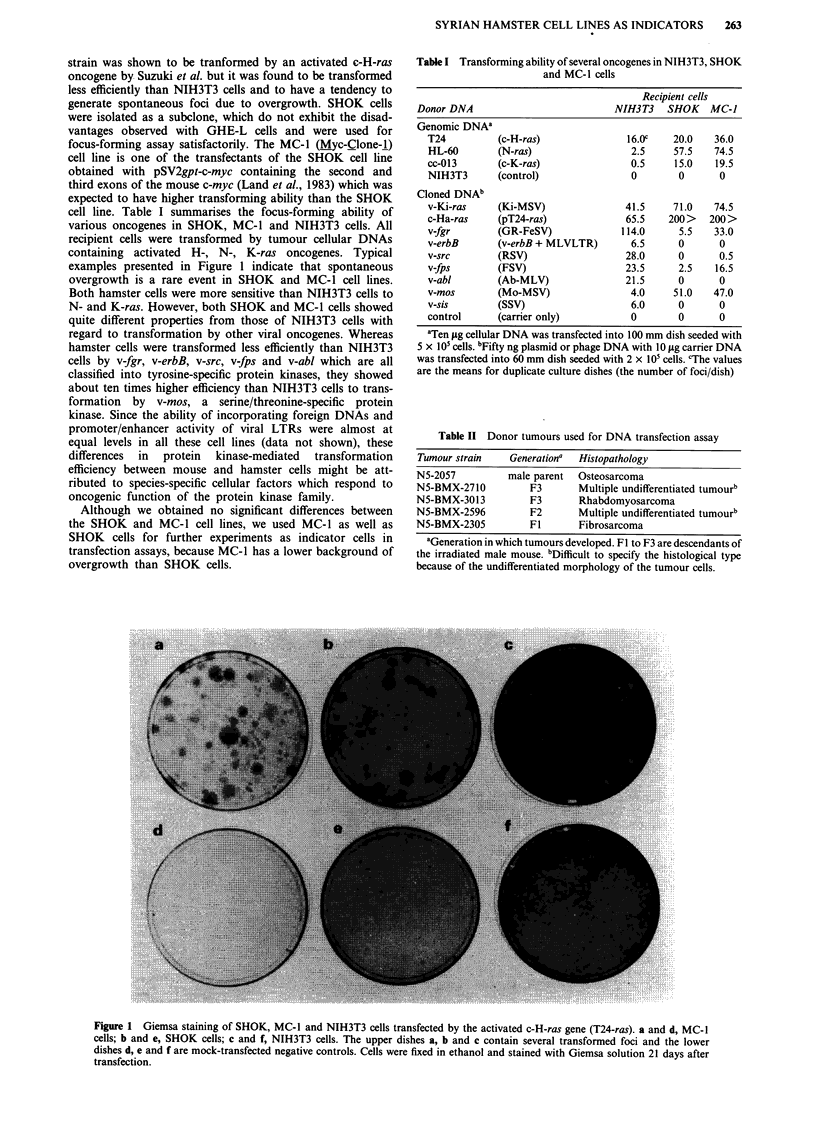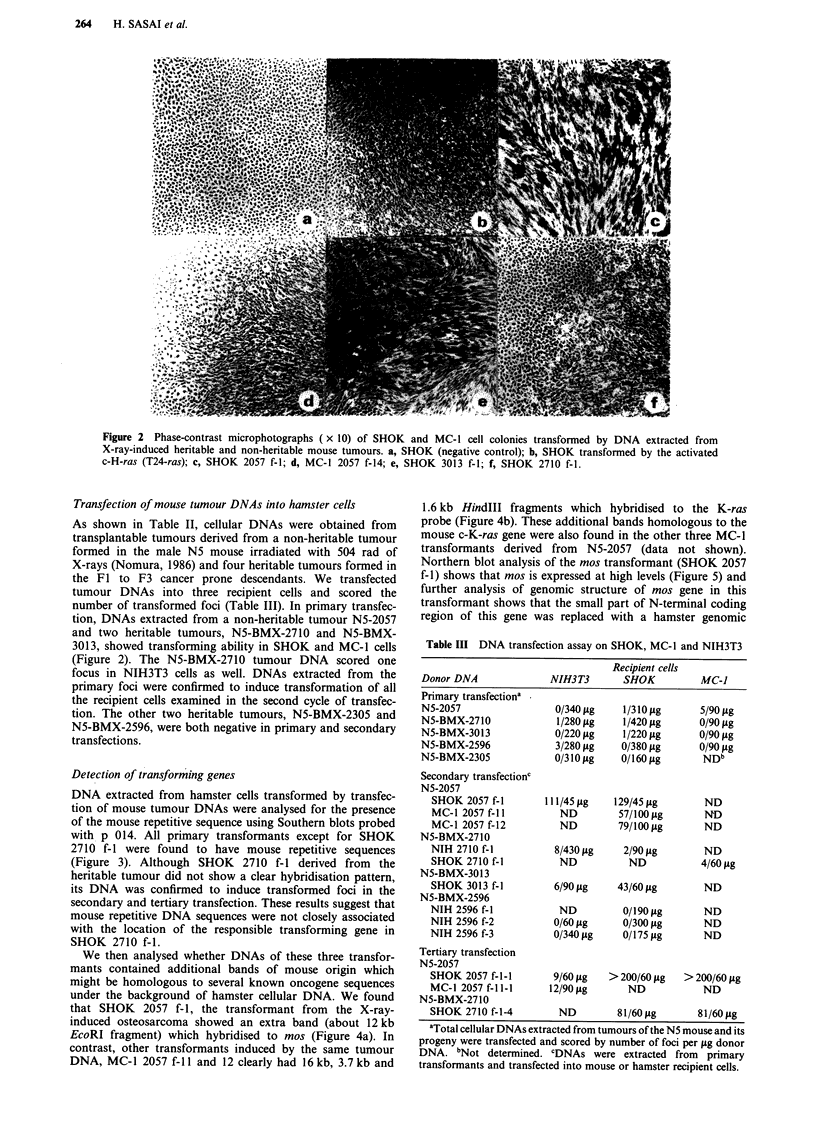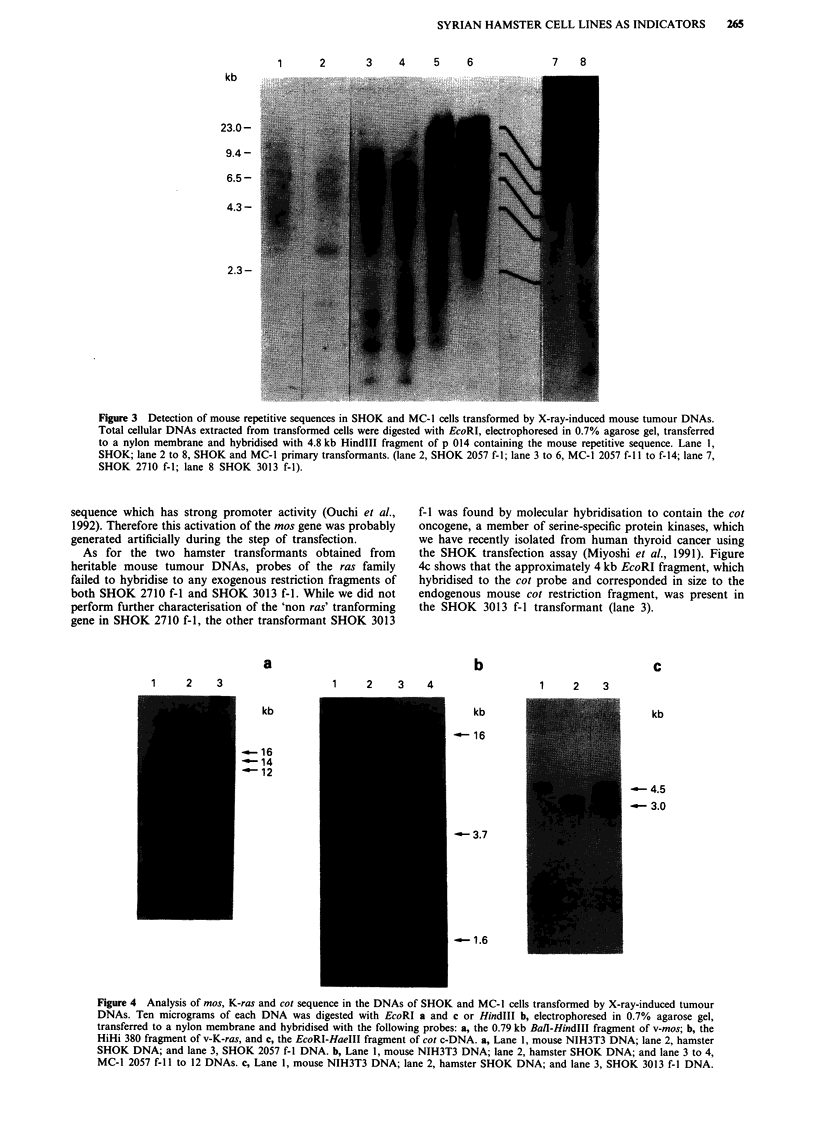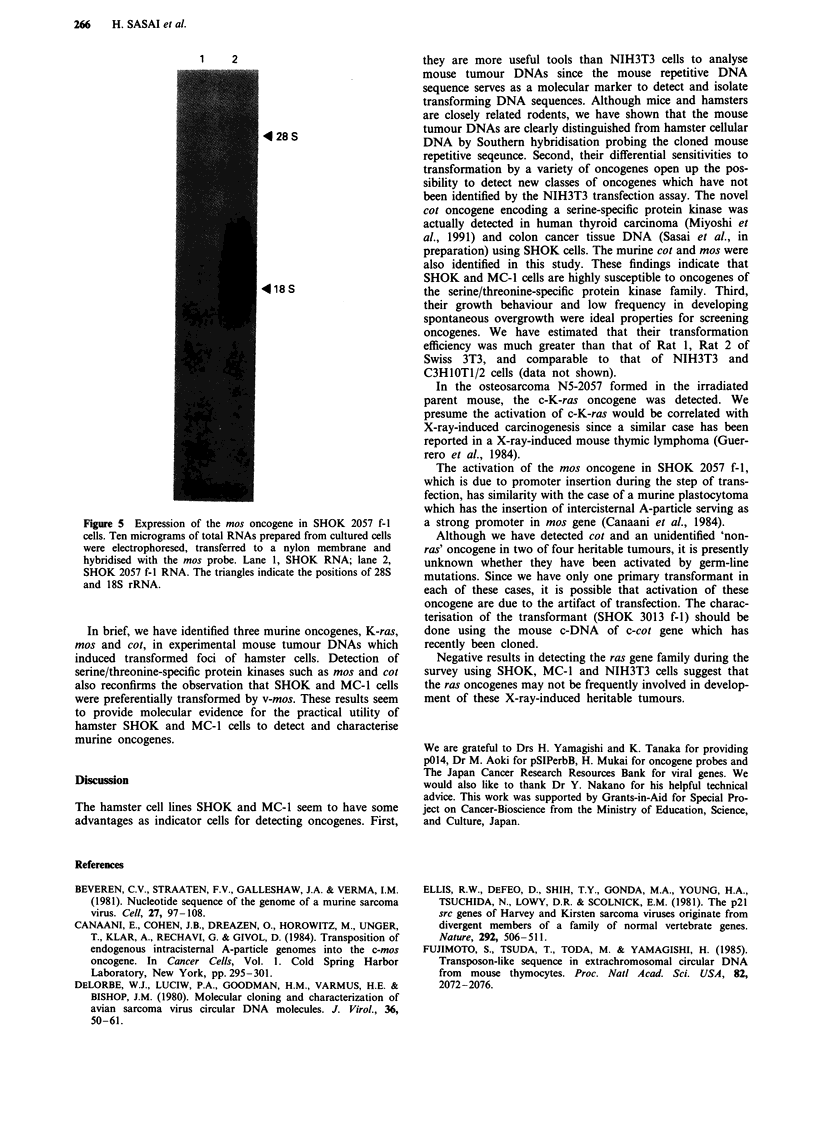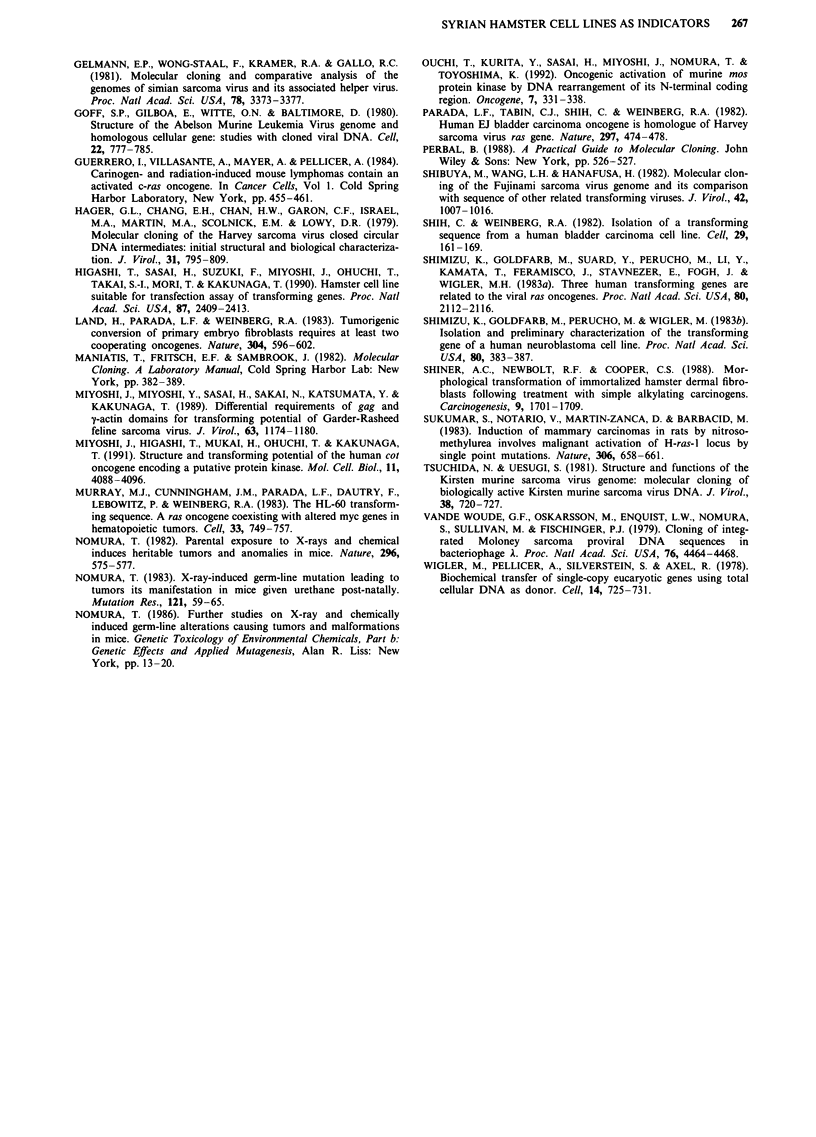# Syrian hamster embryo cell lines useful for detecting transforming genes in mouse tumours: detection of transforming genes in X-ray-related mouse tumours.

**DOI:** 10.1038/bjc.1993.50

**Published:** 1993-02

**Authors:** H. Sasai, T. Higashi, S. Nakamori, J. Miyoshi, F. Suzuki, T. Nomura, T. Kakunaga

**Affiliations:** Department of Oncogene Research, Osaka University, Japan.

## Abstract

**Images:**


*Br. J. Cancer* (1993), **67**, 262–267 © Macmillan Press Ltd., 1993

### Syrian hamster embryo cell lines useful for detecting transforming genes in mouse tumours: detection of transforming genes in X-ray-related mouse tumours

H. Sasai[1], T. Higashi[1], S. Nakamori[1], J. Miyoshi[1], F. Suzuki[2], T. Nomura[3] & T. Kakunaga[1]

[1]*Department of Oncogene Research, Research Institute for Microbial Diseases, Osaka University;* [2]*Division of Radiation Biology, Faculty of Pharmaceutical Sciences, Kanazawa University;* [3]*Department of Radiation Biology, Faculty of Medicine, Osaka University, Japan*

**Summary** The Syrian hamster embryo cell lines, SHOK and MC-1, were used as recipient cells for DNA transfection assay to detect transforming genes in experimental mouse tumours. A mouse repeat sequence was utilised to check whether each transformed focus included mouse genomic DNA in the Hamster background. We investigated five mouse tumours that are related to X-ray radiation, and detected activated c-K-*ras*, c-*mos*, and c-*cot* oncogenes which induced foci of hamster cells. These results show that SHOK and MC-1 cells have unique properties for detecting transforming genes in experimental mouse tumours.

Development of the mouse NIH3T3 transfection assay has led to the identification of several oncogenes in human tumours and animal cells transformed by chemical carcinogens (Parada *et al.*, 1982; Shimizu *et al.*, 1983*a*; Sukumar *et al.*, 1983). Although the roles of such oncogenes in multistage carcinogenesis are not fully understood, we are interested in detecting transforming genes involved in the mouse tumours which are related to X-ray-radiation, to obtain some insight about the role of murine oncogenes. In this experimental system, the descendants of the irradiated mouse N5 strain tend to develop tumours and their susceptibility to tumour formation is inherited as an autosomal dominant trait as if it were induced by germ-line mutation (Nomura, 1982; 1983; 1986). It remains unknown, however, what kind of genetic changes are involved in developing tumours in these mice and whether activated oncogenes are actually present in these tumours or not.

NIH3T3 cells are commonly used for detecting oncogenes because of their high efficiency of transformation by oncogenes. Since mouse-specific repeated DNA sequences do not serve as molecular markers of foreign DNA in NIH3T3 cells, we have tried to find recipient cells of non-mouse origin. Although there are other transfection systems using non-mouse origin cells such as 4DH2 cells (Shiner *et al.*, 1988), their sensitivities against oncogenes are not as good as NIH3T3. We developed the hamster SHOK and MC-1 cell lines whose sensitivity against oncogenes is comparable to NIH3T3 cells.

Here we report the novel properties of these hamster cells as indicator cells for the focus-forming assay and detection of the activated oncogenes in tumours formed in N5 mice exposed to X-rays and cancer prone descendants whose parents' germ line were irradiated with X-rays.

### Materials and methods

### Cell culture

Syrian hamster embryo fibroblast cell lines, SHOK and MC-1 cells, were grown in Dulbecco's modified minimum essential medium (D-MEM) supplemented with 10% foetal calf serum. NIH3T3 cells were grown in D-MEM supplemented with 10% calf serum.

### DNA transfection assay

High molecular weight DNAs extracted from mouse tumours were transfected into NIH3T3, SHOK and MC-1 cells by the calcium phosphate coprecipitation method (Wigler, 1978). After transfection, NIH3T3 cells were maintained in D-MEM supplemented with 5% calf serum, SHOK and MC-1 cells were maintained in D-MEM supplemented with 3% foetal calf serum. The foci were scored 21 days after transfection. DNA samples examined were as follows. Genomic DNA: T24 for c-H-*ras* (Sukumar *et al.*, 1983), HL-60 for N-*ras* (Murray *et al.*, 1983), CC-013 for c-K-*ras* (a NIH3T3 tranformant from colon cancer tissue; Sasai, unpublished work). Cloned plasmid (Tsuchida *et al.*, 1981), pEJ*ras* for c-H-*ras* (Shih *et al.*, 1982), pSV2*neo-fgr* for GR-FeSV (Miyoshi *et al.*, 1989), pZIP*erbB* for AEV (Aoki, unpublished work), pSRA2 for RSV (DeLorbe *et al.*, 1980), λFSV2 for FSV (Shibuya *et al.*, 1982), λAb3 for Abelson MLV (Goff *et al.*, 1980), pMSV-1L for Moloney-MSV (Beveren *et al.*, 1981) and pC60 for SSV (Gelmann *et al.*, 1981).

### Molecular hybridisation of transforming genes

High molecular weight DNAs were digested with restriction endonuclease and fractionated by electrophoresis in 0.7% agarose gell (10 μg DNA/lane) (Maniatis *et al.*, 1982). Ten micrograms of denatured total RNA were fractionated by electrophoresis in 1.0% agarose formaldehyde gels (Perbal, 1988). DNA and RNA samples were transferred to a nylon membrane filter and hybridised to the following probes: p 014 (MIF/Bam/R/B1 superfamily sequence) for the mouse repetitive sequence (Fujimoto *et al.*, 1985), *Hin*dIII fragment (880 bp) of Ha-MSV for Ha-*ras* (Hager *et al.*, 1979), R-fragment (1 kb) for N-*ras* (Shimizu *et al.*, 1983*b*), HiHi 380 fragment for K-*ras* (Ellis *et al.*, 1981), *Bal*I-*Hin*dIII fragment (790 bp) of Mo-MSV-HT-1 for *mos* (Vande Woude *et al.*, 1979) and *Eco*RI-*Hae*III fragment (170 bp) of *cot* c-DNA (Miyoshi *et al.*, 1991). The DNA probes were labelled by the multiprimer extension method to give $10^9$ c.p.m. $\mu g^{-1}$ DNA. The filters were hybridised at 65°C over night in hybridisation buffer containing 6 × SSC, 5 × Denhardt, 0.1% SDS, 20 $\mu g\ ml^{-1}$ salmon sperm DNA. The filters were washed in 2 × SSC, 0.1% SDS at 65°C for 30 min, followed by washing with 0.2 × SSC and 0.1% SDS for 10 min. They were exposed to an X-ray film at − 70°C with an intensifying screen.

### Results

### New cell lines for detecting murine oncogenes

We have established a hamster cell line SHOK (Syrian Hamster Osaka, Kanazawa) from a GHE-L strain of Syrian hamster embryo cells (Higashi *et al.*, 1990). The GHE-L

Correspondence: H. Sasai, Cell Genesys Inc. 344 Lakeside Drive, Foster City, CA 94404, USA.
Received 11 February 1992; and in revised form 15 September 1992

strain was shown to be tranformed by an activated c-H-*ras* oncogene by Suzuki *et al.* but it was found to be transformed less efficiently than NIH3T3 cells and to have a tendency to generate spontaneous foci due to overgrowth. SHOK cells were isolated as a subclone, which do not exhibit the disadvantages observed with GHE-L cells and were used for focus-forming assay satisfactorily. The MC-1 (Myc-Clone-1) cell line is one of the transfectants of the SHOK cell line obtained with pSV2*gpt*-c-*myc* containing the second and third exons of the mouse c-*myc* (Land *et al.*, 1983) which was expected to have higher transforming ability than the SHOK cell line. Table I summarises the focus-forming ability of various oncogenes in SHOK, MC-1 and NIH3T3 cells. All recipient cells were transformed by tumour cellular DNAs containing activated H-, N-, K-*ras* oncogenes. Typical examples presented in Figure 1 indicate that spontaneous overgrowth is a rare event in SHOK and MC-1 cell lines. Both hamster cells were more sensitive than NIH3T3 cells to N- and K-*ras*. However, both SHOK and MC-1 cells showed quite different properties from those of NIH3T3 cells with regard to transformation by other viral oncogenes. Whereas hamster cells were transformed less efficiently than NIH3T3 cells by v-*fgr*, v-*erbB*, v-*src*, v-*fps* and v-*abl* which are all classified into tyrosine-specific protein kinases, they showed about ten times higher efficiency than NIH3T3 cells to transformation by v-*mos*, a serine/threonine-specific protein kinase. Since the ability of incorporating foreign DNAs and promoter/enhancer activity of viral LTRs were almost at equal levels in all these cell lines (data not shown), these differences in protein kinase-mediated transformation efficiency between mouse and hamster cells might be attributed to species-specific cellular factors which respond to oncogenic function of the protein kinase family.

Although we obtained no significant differences between the SHOK and MC-1 cell lines, we used MC-1 as well as SHOK cells for further experiments as indicator cells in transfection assays, because MC-1 has a lower background of overgrowth than SHOK cells.

**Table I** Transforming ability of several oncogenes in NIH3T3, SHOK and MC-1 cells

| *Donor DNA* | | *Recipient cells* *NIH3T3* | *SHOK* | *MC-1* |
|---|---|---|---|---|
| Genomic DNA[a] | | | | |
| T24 | (c-H-*ras*) | 16.0[c] | 20.0 | 36.0 |
| HL-60 | (N-*ras*) | 2.5 | 57.5 | 74.5 |
| cc-013 | (c-K-*ras*) | 0.5 | 15.0 | 19.5 |
| NIH3T3 | (control) | 0 | 0 | 0 |
| Cloned DNA[b] | | | | |
| v-Ki-*ras* | (Ki-MSV) | 41.5 | 71.0 | 74.5 |
| c-Ha-*ras* | (pT24-*ras*) | 65.5 | 200> | 200> |
| v-*fgr* | (GR-FeSV) | 114.0 | 5.5 | 33.0 |
| v-*erbB* | (v-*erbB* + MLVLTR) | 6.5 | 0 | 0 |
| v-*src* | (RSV) | 28.0 | 0 | 0.5 |
| v-*fps* | (FSV) | 23.5 | 2.5 | 16.5 |
| v-*abl* | (Ab-MLV) | 21.5 | 0 | 0 |
| v-*mos* | (Mo-MSV) | 4.0 | 51.0 | 47.0 |
| v-*sis* | (SSV) | 6.0 | 0 | 0 |
| control | (carrier only) | 0 | 0 | 0 |

[a]Ten μg cellular DNA was transfected into 100 mm dish seeded with $5 \times 10^5$ cells. [b]Fifty ng plasmid or phage DNA with 10 μg carrier DNA was transfected into 60 mm dish seeded with $2 \times 10^5$ cells. [c]The values are the means for duplicate culture dishes (the number of foci/dish)

**Table II** Donor tumours used for DNA transfection assay

| *Tumour strain* | *Generation*[a] | *Histopathology* |
|---|---|---|
| N5-2057 | male parent | Osteosarcoma |
| N5-BMX-2710 | F3 | Multiple undifferentiated tumour[b] |
| N5-BMX-3013 | F3 | Rhabdomyosarcoma |
| N5-BMX-2596 | F2 | Multiple undifferentiated tumour[b] |
| N5-BMX-2305 | F1 | Fibrosarcoma |

[a]Generation in which tumours developed. F1 to F3 are descendants of the irradiated male mouse. [b]Difficult to specify the histological type because of the undifferentiated morphology of the tumour cells.

**Figure 1** Giemsa staining of SHOK, MC-1 and NIH3T3 cells transfected by the activated c-H-*ras* gene (T24-*ras*). **a** and **d**, MC-1 cells; **b** and **e**, SHOK cells; **c** and **f**, NIH3T3 cells. The upper dishes **a**, **b** and **c** contain several transformed foci and the lower dishes **d**, **e** and **f** are mock-transfected negative controls. Cells were fixed in ethanol and stained with Giemsa solution 21 days after transfection.

**Figure 2** Phase-contrast microphotographs ( × 10) of SHOK and MC-1 cell colonies transformed by DNA extracted from X-ray-induced heritable and non-heritable mouse tumours. **a**, SHOK (negative control); **b**, SHOK transformed by the activated c-H-*ras* (T24-*ras*); **c**, SHOK 2057 f-1; **d**, MC-1 2057 f-14; **e**, SHOK 3013 f-1; **f**, SHOK 2710 f-1.

*Transfection of mouse tumour DNAs into hamster cells*

As shown in Table II, cellular DNAs were obtained from transplantable tumours derived from a non-heritable tumour formed in the male N5 mouse irradiated with 504 rad of X-rays (Nomura, 1986) and four heritable tumours formed in the F1 to F3 cancer prone descendants. We transfected tumour DNAs into three recipient cells and scored the number of transformed foci (Table III). In primary transfection, DNAs extracted from a non-heritable tumour N5-2057 and two heritable tumours, N5-BMX-2710 and N5-BMX-3013, showed transforming ability in SHOK and MC-1 cells (Figure 2). The N5-BMX-2710 tumour DNA scored one focus in NIH3T3 cells as well. DNAs extracted from the primary foci were confirmed to induce transformation of all the recipient cells examined in the second cycle of transfection. The other two heritable tumours, N5-BMX-2305 and N5-BMX-2596, were both negative in primary and secondary transfections.

*Detection of transforming genes*

DNA extracted from hamster cells transformed by transfection of mouse tumour DNAs were analysed for the presence of the mouse repetitive sequence using Southern blots probed with p 014. All primary transformants except for SHOK 2710 f-1 were found to have mouse repetitive sequences (Figure 3). Although SHOK 2710 f-1 derived from the heritable tumour did not show a clear hybridisation pattern, its DNA was confirmed to induce transformed foci in the secondary and tertiary transfection. These results suggest that mouse repetitive DNA sequences were not closely associated with the location of the responsible transforming gene in SHOK 2710 f-1.

We then analysed whether DNAs of these three transformants contained additional bands of mouse origin which might be homologous to several known oncogene sequences under the background of hamster cellular DNA. We found that SHOK 2057 f-1, the transformant from the X-ray-induced osteosarcoma showed an extra band (about 12 kb *Eco*RI fragment) which hybridised to *mos* (Figure 4a). In contrast, other transformants induced by the same tumour DNA, MC-1 2057 f-11 and 12 clearly had 16 kb, 3.7 kb and 1.6 kb *Hin*dIII fragments which hybridised to the K-*ras* probe (Figure 4b). These additional bands homologous to the mouse c-K-*ras* gene were also found in the other three MC-1 transformants derived from N5-2057 (data not shown). Northern blot analysis of the *mos* transformant (SHOK 2057 f-1) shows that *mos* is expressed at high levels (Figure 5) and further analysis of genomic structure of *mos* gene in this transformant shows that the small part of N-terminal coding region of this gene was replaced with a hamster genomic

**Table III** DNA transfection assay on SHOK, MC-1 and NIH3T3

| *Donor DNA* | | *Recipient cells* | |
|---|---|---|---|
| | *NIH3T3* | *SHOK* | *MC-1* |
| Primary transfection[a] | | | |
| N5-2057 | 0/340 μg | 1/310 μg | 5/90 μg |
| N5-BMX-2710 | 1/280 μg | 1/420 μg | 0/90 μg |
| N5-BMX-3013 | 0/220 μg | 1/220 μg | 0/90 μg |
| N5-BMX-2596 | 3/280 μg | 0/380 μg | 0/90 μg |
| N5-BMX-2305 | 0/310 μg | 0/160 μg | ND[b] |
| Secondary transfection[c] | | | |
| N5-2057 | | | |
| SHOK 2057 f-1 | 111/45 μg | 129/45 μg | ND |
| MC-1 2057 f-11 | ND | 57/100 μg | ND |
| MC-1 2057 f-12 | ND | 79/100 μg | ND |
| N5-BMX-2710 | | | |
| NIH 2710 f-1 | 8/430 μg | 2/90 μg | ND |
| SHOK 2710 f-1 | ND | ND | 4/60 μg |
| N5-BMX-3013 | | | |
| SHOK 3013 f-1 | 6/90 μg | 43/60 μg | ND |
| N5-BMX-2596 | | | |
| NIH 2596 f-1 | ND | 0/190 μg | ND |
| NIH 2596 f-2 | 0/60 μg | 0/300 μg | ND |
| NIH 2596 f-3 | 0/340 μg | 0/175 μg | ND |
| Tertiary transfection | | | |
| N5-2057 | | | |
| SHOK 2057 f-1-1 | 9/60 μg | >200/60 μg | >200/60 μg |
| MC-1 2057 f-11-1 | 12/90 μg | ND | ND |
| N5-BMX-2710 | | | |
| SHOK 2710 f-1-4 | ND | 81/60 μg | 81/60 μg |

[a]Total cellular DNAs extracted from tumours of the N5 mouse and its progeny were transfected and scored by number of foci per μg donor DNA. [b]Not determined. [c]DNAs were extracted from primary transformants and transfected into mouse or hamster recipient cells.

**Figure 3** Detection of mouse repetitive sequences in SHOK and MC-1 cells transformed by X-ray-induced mouse tumour DNAs. Total cellular DNAs extracted from transformed cells were digested with *Eco*RI, electrophoresed in 0.7% agarose gel, transferred to a nylon membrane and hybridised with 4.8 kb HindIII fragment of p 014 containing the mouse repetitive sequence. Lane 1, SHOK; lane 2 to 8, SHOK and MC-1 primary transformants. (lane 2, SHOK 2057 f-1; lane 3 to 6, MC-1 2057 f-11 to f-14; lane 7, SHOK 2710 f-1; lane 8 SHOK 3013 f-1).

sequence which has strong promoter activity (Ouchi *et al.*, 1992). Therefore this activation of the *mos* gene was probably generated artificially during the step of transfection.

As for the two hamster transformants obtained from heritable mouse tumour DNAs, probes of the *ras* family failed to hybridise to any exogenous restriction fragments of both SHOK 2710 f-1 and SHOK 3013 f-1. While we did not perform further characterisation of the 'non *ras*' tranforming gene in SHOK 2710 f-1, the other transformant SHOK 3013 f-1 was found by molecular hybridisation to contain the *cot* oncogene, a member of serine-specific protein kinases, which we have recently isolated from human thyroid cancer using the SHOK transfection assay (Miyoshi *et al.*, 1991). Figure 4c shows that the approximately 4 kb *Eco*RI fragment, which hybridised to the *cot* probe and corresponded in size to the endogenous mouse *cot* restriction fragment, was present in the SHOK 3013 f-1 transformant (lane 3).

**Figure 4** Analysis of *mos*, K-*ras* and *cot* sequence in the DNAs of SHOK and MC-1 cells transformed by X-ray-induced tumour DNAs. Ten micrograms of each DNA was digested with *Eco*RI **a** and **c** or *Hin*dIII **b**, electrophoresed in 0.7% agarose gel, transferred to a nylon membrane and hybridised with the following probes: **a**, the 0.79 kb *Bal*I-*Hin*dIII fragment of v-*mos*; **b**, the HiHi 380 fragment of v-K-*ras*, and **c**, the *Eco*RI-*Hae*III fragment of *cot* c-DNA. **a**, Lane 1, mouse NIH3T3 DNA; lane 2, hamster SHOK DNA; and lane 3, SHOK 2057 f-1 DNA. **b**, Lane 1, mouse NIH3T3 DNA; lane 2, hamster SHOK DNA; and lane 3 to 4, MC-1 2057 f-11 to 12 DNAs. **c**, Lane 1, mouse NIH3T3 DNA; lane 2, hamster SHOK DNA; and lane 3, SHOK 3013 f-1 DNA.

**Figure 5** Expression of the *mos* oncogene in SHOK 2057 f-1 cells. Ten micrograms of total RNAs prepared from cultured cells were electrophoresed, transferred to a nylon membrane and hybridised with the *mos* probe. Lane 1, SHOK RNA; lane 2, SHOK 2057 f-1 RNA. The triangles indicate the positions of 28S and 18S rRNA.

In brief, we have identified three murine oncogenes, K-*ras*, *mos* and *cot*, in experimental mouse tumour DNAs which induced transformed foci of hamster cells. Detection of serine/threonine-specific protein kinases such as *mos* and *cot* also reconfirms the observation that SHOK and MC-1 cells were preferentially transformed by v-*mos*. These results seem to provide molecular evidence for the practical utility of hamster SHOK and MC-1 cells to detect and characterise murine oncogenes.

### Discussion

The hamster cell lines SHOK and MC-1 seem to have some advantages as indicator cells for detecting oncogenes. First, they are more useful tools than NIH3T3 cells to analyse mouse tumour DNAs since the mouse repetitive DNA sequence serves as a molecular marker to detect and isolate transforming DNA sequences. Although mice and hamsters are closely related rodents, we have shown that the mouse tumour DNAs are clearly distinguished from hamster cellular DNA by Southern hybridisation probing the cloned mouse repetitive seqeunce. Second, their differential sensitivities to transformation by a variety of oncogenes open up the possibility to detect new classes of oncogenes which have not been identified by the NIH3T3 transfection assay. The novel *cot* oncogene encoding a serine-specific protein kinase was actually detected in human thyroid carcinoma (Miyoshi *et al.*, 1991) and colon cancer tissue DNA (Sasai *et al.*, in preparation) using SHOK cells. The murine *cot* and *mos* were also identified in this study. These findings indicate that SHOK and MC-1 cells are highly susceptible to oncogenes of the serine/threonine-specific protein kinase family. Third, their growth behaviour and low frequency in developing spontaneous overgrowth were ideal properties for screening oncogenes. We have estimated that their transformation efficiency was much greater than that of Rat 1, Rat 2 of Swiss 3T3, and comparable to that of NIH3T3 and C3H10T1/2 cells (data not shown).

In the osteosarcoma N5-2057 formed in the irradiated parent mouse, the c-K-*ras* oncogene was detected. We presume the activation of c-K-*ras* would be correlated with X-ray-induced carcinogenesis since a similar case has been reported in a X-ray-induced mouse thymic lymphoma (Guerrero *et al.*, 1984).

The activation of the *mos* oncogene in SHOK 2057 f-1, which is due to promoter insertion during the step of transfection, has similarity with the case of a murine plastocytoma which has the insertion of intercisternal A-particle serving as a strong promoter in *mos* gene (Canaani *et al.*, 1984).

Although we have detected *cot* and an unidentified 'non-*ras*' oncogene in two of four heritable tumours, it is presently unknown whether they have been activated by germ-line mutations. Since we have only one primary transformant in each of these cases, it is possible that activation of these oncogene are due to the artifact of transfection. The characterisation of the transformant (SHOK 3013 f-1) should be done using the mouse c-DNA of c-*cot* gene which has recently been cloned.

Negative results in detecting the *ras* gene family during the survey using SHOK, MC-1 and NIH3T3 cells suggest that the *ras* oncogenes may not be frequently involved in development of these X-ray-induced heritable tumours.

We are grateful to Drs H. Yamagishi and K. Tanaka for providing p014, Dr M. Aoki for pSIPerbB, H. Mukai for oncogene probes and The Japan Cancer Research Resources Bank for viral genes. We would also like to thank Dr Y. Nakano for his helpful technical advice. This work was supported by Grants-in-Aid for Special Project on Cancer-Bioscience from the Ministry of Education, Science, and Culture, Japan.

### References

BEVEREN, C.V., STRAATEN, F.V., GALLESHAW, J.A. & VERMA, I.M. (1981). Nucleotide sequence of the genome of a murine sarcoma virus. *Cell*, **27**, 97–108.

CANAANI, E., COHEN, J.B., DREAZEN, O., HOROWITZ, M., UNGER, T., KLAR, A., RECHAVI, G. & GIVOL, D. (1984). Transposition of endogenous intracisternal A-particle genomes into the c-*mos* oncogene. In *Cancer Cells*, Vol. 1. Cold Spring Harbor Laboratory, New York, pp. 295–301.

DELORBE, W.J., LUCIW, P.A., GOODMAN, H.M., VARMUS, H.E. & BISHOP, J.M. (1980). Molecular cloning and characterization of avian sarcoma virus circular DNA molecules. *J. Virol.*, **36**, 50–61.

ELLIS, R.W., DEFEO, D., SHIH, T.Y., GONDA, M.A., YOUNG, H.A., TSUCHIDA, N., LOWY, D.R. & SCOLNICK, E.M. (1981). The p21 *src* genes of Harvey and Kirsten sarcoma viruses originate from divergent members of a family of normal vertebrate genes. *Nature*, **292**, 506–511.

FUJIMOTO, S., TSUDA, T., TODA, M. & YAMAGISHI, H. (1985). Transposon-like sequence in extrachromosomal circular DNA from mouse thymocytes. *Proc. Natl Acad. Sci. USA*, **82**, 2072–2076.

GELMANN, E.P., WONG-STAAL, F., KRAMER, R.A. & GALLO, R.C. (1981). Molecular cloning and comparative analysis of the genomes of simian sarcoma virus and its associated helper virus. *Proc. Natl Acad. Sci. USA*, **78**, 3373–3377.

GOFF, S.P., GILBOA, E., WITTE, O.N. & BALTIMORE, D. (1980). Structure of the Abelson Murine Leukemia Virus genome and homologous cellular gene: studies with cloned viral DNA. *Cell*, **22**, 777–785.

GUERRERO, I., VILLASANTE, A., MAYER, A. & PELLICER, A. (1984). Carinogen- and radiation-induced mouse lymphomas contain an activated c-*ras* oncogene. In *Cancer Cells*, Vol 1. Cold Spring Harbor Laboratory, New York, pp. 455–461.

HAGER, G.L., CHANG, E.H., CHAN, H.W., GARON, C.F., ISRAEL, M.A., MARTIN, M.A., SCOLNICK, E.M. & LOWY, D.R. (1979). Molecular cloning of the Harvey sarcoma virus closed circular DNA intermediates: initial structural and biological characterization. *J. Virol.*, **31**, 795–809.

HIGASHI, T., SASAI, H., SUZUKI, F., MIYOSHI, J., OHUCHI, T., TAKAI, S.-I., MORI, T. & KAKUNAGA, T. (1990). Hamster cell line suitable for transfection assay of transforming genes. *Proc. Natl Acad. Sci. USA*, **87**, 2409–2413.

LAND, H., PARADA, L.F. & WEINBERG, R.A. (1983). Tumorigenic conversion of primary embryo fibroblasts requires at least two cooperating oncogenes. *Nature*, **304**, 596–602.

MANIATIS, T., FRITSCH, E.F. & SAMBROOK, J. (1982). *Molecular Cloning. A Laboratory Manual*, Cold Spring Harbor Lab: New York, pp. 382–389.

MIYOSHI, J., MIYOSHI, Y., SASAI, H., SAKAI, N., KATSUMATA, Y. & KAKUNAGA, T. (1989). Differential requirements of *gag* and γ-actin domains for transforming potential of Garder-Rasheed feline sarcoma virus. *J. Virol.*, **63**, 1174–1180.

MIYOSHI, J., HIGASHI, T., MUKAI, H., OHUCHI, T. & KAKUNAGA, T. (1991). Structure and transforming potential of the human *cot* oncogene encoding a putative protein kinase. *Mol. Cell. Biol.*, **11**, 4088–4096.

MURRAY, M.J., CUNNINGHAM, J.M., PARADA, L.F., DAUTRY, F., LEBOWITZ, P. & WEINBERG, R.A. (1983). The HL-60 transforming sequence. A *ras* oncogene coexisting with altered myc genes in hematopoietic tumors. *Cell*, **33**, 749–757.

NOMURA, T. (1982). Parental exposure to X-rays and chemical induces heritable tumors and anomalies in mice. *Nature*, **296**, 575–577.

NOMURA, T. (1983). X-ray-induced germ-line mutation leading to tumors its manifestation in mice given urethane post-natally. *Mutation Res.*, **121**, 59–65.

NOMURA, T. (1986). Further studies on X-ray and chemically induced germ-line alterations causing tumors and malformations in mice. *Genetic Toxicology of Environmental Chemicals, Part b: Genetic Effects and Applied Mutagenesis*, Alan R. Liss: New York, pp. 13–20.

OUCHI, T., KURITA, Y., SASAI, H., MIYOSHI, J., NOMURA, T. & TOYOSHIMA, K. (1992). Oncogenic activation of murine *mos* protein kinase by DNA rearrangement of its N-terminal coding region. *Oncogene*, **7**, 331–338.

PARADA, L.F., TABIN, C.J., SHIH, C. & WEINBERG, R.A. (1982). Human EJ bladder carcinoma oncogene is homologue of Harvey sarcoma virus *ras* gene. *Nature*, **297**, 474–478.

PERBAL, B. (1988). *A Practical Guide to Molecular Cloning*. John Wiley & Sons: New York, pp. 526–527.

SHIBUYA, M., WANG, L.H. & HANAFUSA, H. (1982). Molecular cloning of the Fujinami sarcoma virus genome and its comparison with sequence of other related transforming viruses. *J. Virol.*, **42**, 1007–1016.

SHIH, C. & WEINBERG, R.A. (1982). Isolation of a transforming sequence from a human bladder carcinoma cell line. *Cell*, **29**, 161–169.

SHIMIZU, K., GOLDFARB, M., SUARD, Y., PERUCHO, M., LI, Y., KAMATA, T., FERAMISCO, J., STAVNEZER, E., FOGH, J. & WIGLER, M.H. (1983*a*). Three human transforming genes are related to the viral *ras* oncogenes. *Proc. Natl Acad. Sci. USA*, **80**, 2112–2116.

SHIMIZU, K., GOLDFARB, M., PERUCHO, M. & WIGLER, M. (1983*b*). Isolation and preliminary characterization of the transforming gene of a human neuroblastoma cell line. *Proc. Natl Acad. Sci. USA*, **80**, 383–387.

SHINER, A.C., NEWBOLT, R.F. & COOPER, C.S. (1988). Morphological transformation of immortalized hamster dermal fibroblasts following treatment with simple alkylating carcinogens. *Carcinogenesis*, **9**, 1701–1709.

SUKUMAR, S., NOTARIO, V., MARTIN-ZANCA, D. & BARBACID, M. (1983). Induction of mammary carcinomas in rats by nitrosomethylurea involves malignant activation of H-*ras*-1 locus by single point mutations. *Nature*, **306**, 658–661.

TSUCHIDA, N. & UESUGI, S. (1981). Structure and functions of the Kirsten murine sarcoma virus genome: molecular cloning of biologically active Kirsten murine sarcoma virus DNA. *J. Virol.*, **38**, 720–727.

VANDE WOUDE, G.F., OSKARSSON, M., ENQUIST, L.W., NOMURA, S., SULLIVAN, M. & FISCHINGER, P.J. (1979). Cloning of integrated Moloney sarcoma proviral DNA sequences in bacteriophage λ. *Proc. Natl Acad. Sci. USA*, **76**, 4464–4468.

WIGLER, M., PELLICER, A., SILVERSTEIN, S. & AXEL, R. (1978). Biochemical transfer of single-copy eucaryotic genes using total cellular DNA as donor. *Cell*, **14**, 725–731.